# Chimeric antigen receptor T cells targeting PD-L1 suppress tumor growth

**DOI:** 10.1186/s40364-020-00198-0

**Published:** 2020-06-03

**Authors:** Le Qin, Ruocong Zhao, Dongmei Chen, Xinru Wei, Qiting Wu, Youguo Long, Zhiwu Jiang, Yangqiu Li, Haipeng Wu, Xuchao Zhang, Yilong Wu, Shuzhong Cui, Wei Wei, Huihui Yao, Zixia Liu, Su Cao, Yao Yao, Zhenfeng Zhang, Peng Li

**Affiliations:** 1 State Key Laboratory of Respiratory Disease, Guangdong Provincial Key Laboratory of Stem Cell and Regenerative Medicine, Guangzhou Institutes of Biomedicine and Health, Chinese Academy of Sciences, Guangzhou, China; 2 University of Chinese Academy of Sciences, Beijing, China; 3 Institute of Hematology, Medical College, Jinan University, Guangzhou, China; 4 Guangdong Zhaotai InVivo Biomedicine Co. Ltd., Guangzhou, China; 5 Guangdong Lung Cancer Institute, Medical Research Center, Guangdong General Hospital, Guangdong Academy of Medical Sciences, Guangzhou, China; 6 Affiliated Cancer Hospital & Institute of Guangzhou Medical University, Guangzhou, China; 7 Guangdong Cord Blood Bank, Guangzhou, China; 8 The 91th Military Hospital, Jiaozuo, China; 9 Department of Radiology, The Second Affiliated Hospital of Guangzhou Medical University, Guangzhou, China; 10 Guangzhou Regenerative Medicine and Health Guangdong Laboratory (GRMH-GD), Guangzhou, China

**Keywords:** PD-L1, CAR-T cells, Mesothelin, PDX

## Abstract

**Background:**

Chimeric antigen receptor T cells (CAR-T cells) therapy has been well recognized for treating B cell-derived malignancy. However, the efficacy of CAR-T cells against solid tumors remains dissatisfactory, partially due to the heterogeneity of solid tumors and T cell exhaustion in tumor microenvironment. PD-L1 is up-regulated in multiple solid tumors, resulting in T cell exhaustion upon binding to its receptor PD-1.

**Methods:**

Here, we designed a dominant-negative form of PD-1, dPD1z, a vector containing the extracellular and transmembrane regions of human PD-1, and a CAR vector against PD-L1, CARPD-L1z, a vector employs a high-affinity single-chain variable fragment (scFv) against human PD-L1. These two vectors shared the same intracellular structure, including 4-1BB and TLR2 co-stimulatory domains, and the CD3ζ signaling domain.

**Results:**

dPD1z T and CARPD-L1z T cells efficiently lysed PD-L1^+^ tumor cells and had enhanced cytokine secretion in vitro and suppressed the growth of non-small cell lung cancer (NSCLC), gastric cancer and hepatoma carcinoma in patient-derived xenograft (PDX). However, the combination of anti-mesothelin CAR-T cells (CARMSLNz T) with dPD1z T or CARPD-L1z T cells did not repress tumor growth synergistically in PDX, as CARMSLNz T cells upregulated PD-L1 expression upon activation and were subsequently attacked by dPD1z T or CARPD-L1z T cells.

**Conclusions:**

In conclusion, we demonstrate CAR-T cells targeting PD-L1 were effective for suppressing the growth of multiple types of solid tumors in PDX models though their safety needs to be carefully examined.

## Background

Recently, chimeric antigen receptor T cells (CAR-T cells) have emerged as a promising therapy for treating B cell-derived malignancy [1, 2]. Two CAR-T cell products have been approved by the FDA to treat B-cell leukemia and lymphoma [3–6]. CAR-T cells against tumor-specific antigens (TSA), including mesothelin (MSLN) and glypican 3 (GPC3) are being actively tested for treating non-small-cell lung cancer (NSCLC) and hepatocellular carcinoma, respectively [7–19]. However, the effects of CAR-T cells against solid tumors are far from being satisfactory, partially due to the heterogeneity of solid tumors and T cell exhaustion in tumor microenvironment [20–22].

PD-1, a well-characterized immune checkpoint molecule, plays pivotal roles in regulating T cell function. PD-1 upregulation is associated with T cell exhaustion that inhibits T cell functions upon binding to its ligands, such as PD-L1 and PD-L2 [23]. PD-L1 is widely expressed in various solid tumors [24–26]. Its expression is influenced by IFN-γ and is correlated with poor prognosis [27, 28]. Antibody-based checkpoint blockade targeting PD-L1 or its receptor PD-1 has revolutionized the clinical management of multiple cancers [29–35]. Moreover, the ablation of PD-1 improves the persistence of TCR-T cells in patients with solid tumors [36]. Besides diminishing the PD-1/PD-L1 axis, a chimeric switch-receptor comprising the truncated extracellular domain of PD-1 and the transmembrane and cytoplasmic signaling domains of CD28 augments the efficacy of CAR-T cells in solid tumors [37–39]. Nevertheless, modified T cells in these studies still rely on transgenic TCR or CAR or their own TCR to recognize TSA in tumors. Heterogeneous cancer cells thus can escape attacks of these T cells by reducing TSA, leading to tumor recurrence.

Here, we designed a dominant-negative form of PD-1, dPD1z, which does not only contain the extracellular and transmembrane domains of human PD-1, co-stimulation domains but also CD3ζ signaling domain. Different from the chimeric switch-receptor targeting PD-1 that lack CD3ζ signaling domain, dPD1z T cells were not suppressed by PD-L1 but lysed PD-L1 positive tumor cells in vitro and eliminated multiple types of tumors in xenograft.

## Methods

### Lentiviral vectors construction

The extracellular and transmembrane portions of dPD1z derived from PD1 receptor (Uniprot Entry Q15116, amino acids (aa) 1–191), and CARPD-L1z contained a high-affinity anti-PD-L1 scFv that derived from Atezolizumab. The cytoplasmic domains of dPD1z and CARPD-L1z both contain 4-1BB (Uniprot Entry Q07011, aa 214–255), TLR2 (Uniprot Entry O060603, aa 636–784) and the CD3ζ (Uniprot Entry P20963, aa 52–164). The scFvs of CARMSLNz and CAR19z derived from SS1 and FMC63 respectively, in tandem with CD28 (Uniprot Entry P10747, aa 180–220), TLR2 and CD3ζ. The structure of CAR19BBz is same as CAR19z except for that CD28 co-stimulatory domain is replaced with 4-1BB co-stimulatory domain. GL vector contained the firefly luciferase reporter gene (GenBank ABA41653.1, aa 1–550) and eGFP reporter genes (GenBank YP_009062989.1, aa 1–239) linked through 2A peptide. The gene of MSLN (GenBank NP_001170826.1, aa 1–622) was overexpressed in H460GL generated H460-MSLNGL. The uPD-L1 contained the full-length gene of PD-L1 (GenBank NP_001254635.1, aa 1–176) and labeled with a truncated CD19. DNA sequences were synthesized by Genscript (Nanjing) Co., Ltd. (Nanjing, China) and cloned into the second-generation lentiviral vector pWPXLd.

### Lentivirus manufacture

Lentivirus particles were produced in HEK-293 T cells via PEI MAX 40 K (Polyscience, 24,765–1) transfection. HEK-293 T cells were co-transfected with the pWPXLd-based lentiviral plasmid and two packaging plasmids, psPAX2 and pMD2.G. Lentivirus-containing supernatants were harvested at 48 and 72 h post-transduction and filtered through a 0.45-μm filter.

### CAR T cells manufacture

Peripheral blood mononuclear cells (PBMCs) were isolated from healthy donors using Lymphoprep (StemCell Technologies, 07851). T cells were negatively selected from PBMCs using a Pan T cell isolation kit (MiltenyiBiotec, 130–096-535) and activated using microbeads coated with anti-human CD2, anti-human CD3 and anti-human CD28 antibodies (MiltenyiBiotec, 130–091-441) for 2 days in RPMI-1640 medium supplemented with 10% fetal bovine serum (FBS) and 1% penicillin/streptomycin. On day 2 post-activation, T cells were transduced with lentiviral supernatants in the presence of 8 μg/ml polybrene (Sigma-Aldrich, TR-1003-G). Twelve hours post-transduction, T cells were cultured in fresh medium containing IL-2 (300 IU/ml), subsequently, fresh medium was added every 2–3 days to maintain cell density within the range of 0.5–1 × 10^6^/ml. Healthy PBMC donors provided informed consent for the use of their samples for research purposes, and all procedures were approved by the Research Ethics Board of the Guangzhou Institutes of Biomedicine and Health (GIBH).

### Cells and culture conditions

HEK-293 T cells were maintained in Dulbecco’s modified Eagle’s medium (DMEM) (Gibco, C11995500BT). H460 (human large cell lung cancer), MKN-28 (human gastric carcinoma), SMMC-7721 (human hepatoma carcinoma), HeLa (human cervical cancer) and NALM6 (CD19^+^ acute lymphoblastic leukemia) cell lines were obtained from ATCC and maintained in RPMI-1640 (Gibco, C11975500BT). GL-expressing cell lines were generated through transduction of the parental cell line with a lentiviral supernatant containing GL and were sorted for GFP expression on a FACS Aria TM cell sorter (BD Biosciences). DMEM and RPMI-1640 medium were supplemented with 10% heat-inactivated FBS (Vigonob, XC6936T) and 1% penicillin/streptomycin. All cells were cultured at 37 °C in an atmosphere of 5% carbon dioxide. Atezolizumab (AZ) is a humanized anti-PD-L1 monoclonal antibody (Selleck).

### Flow cytometry

Flow cytometry was performed on a BD LSRFortessa cytometer, and data were analyzed using FlowJo software. The antibodies used, including anti-human CD3-PE-cyanine 7 (clone: UCHT1), anti-human CD4-APC (clone: GK1.5), anti-human CD4-APCcy7 (clone: GK1.5), anti-human CD8-PE (clone: 53–6.7), anti-human CD8-Pecpcy5.5 (clone: 53–6.7), anti-human CD25-PE (clone: PC61.5), anti-human CD69-APC (clone: H1.2F3), anti-human CD19-APC (clone: 1D3) and anti-human PD-L1-APC (clone: M1H1) were purchased from eBioscience. Anti-human Mesothelin-PE (clone: sc-33,672) was purchased from Santa Cruz Biotechnology. All FACS staining was performed on ice for 30 min, and cells were then washed with PBS containing 1% FBS before cell cytometry. PB, spleen and tumor samples from xenograft mice were treated with a red blood cell lysis buffer (Biolegend), and the cells were stained with the corresponding antibodies.

### Cytotoxicity assays

The target cells H460GL, MKN-28GL, SMMC-7721GL, HeLaGL and H460-MSLNGL (10^4 cell/well) were incubated with CAR T or negative control T cells at the indicated ratios in triplicate wells of U-bottomed 96-well plates. Target cell viability was monitored 24 h later by adding 100 μl/well of the substrate D-Luciferin (potassium salt) (YEASEN, 40901ES03) at 150 μg/ml. Background luminescence was negligible (< 1% of the signal from the wells with only target cells). The viability percentage (%) was equal to the experimental signal/maximal signal× 100, and the lysis percentage was equal to the 100–viability percentage.

### Cytokine release assays

Enzyme-linked immunosorbent assay (ELISA) kits for IL-2, IFN-γ, TNF-α and GM-CSF were purchased from eBioscience, and all ELISAs were performed according to the manufacturer’s protocols. T cells were co-cultured with target cells at a 1:1 E/T ratio for 24 h, then the culture supernatants were collected and analyzed by ELISA kits.

### Quantitative real-time PCR

mRNA was extracted from cells with TRIzol reagent (Thermo Fisher, 15,596,018) and reverse-transcribed into cDNA using a PrimeScript™ RT reagent kit (Takara, RR047A). All reactions were performed with TransStart Tip Green qPCR SuperMix (TransGene, AQ142–11) on a Bio-Rad CFX96 real-time PCR machine using the following primers: human PD-L1 forward, 5′–CCTACTGGCATTTGCTGAACGCAT-3′, and human PD-L1 reverse, 5′-ACCATAGCTGATCATGCAGCGGTA-3′.

### Xenograft models and in vivo assessment

Animal experiments were performed in the Laboratory Animal Center of the GIBH, and all animal procedures were approved by the Animal Welfare Committee of GIBH. All protocols were approved by the relevant institutional animal care and use committee (IACUC). All mice were maintained in specific pathogen-free (SPF)-grade cages and provided autoclaved food and water. To develop the lung cancer cell line xenograft models, 5 × 10^5^ H460GL cells in 200 μl of PBS were injected subcutaneously into the right flanks of NOD-*SCID-IL2Rg−/−*(NSI) mice aged 6–8 weeks. Ten days after tumor cell transplantation, 5 × 10^6^ CAR T cells were injected through the tail vein of mice. Tumors were measured at indicated days with a caliper to determine the subcutaneous growth rate.

To develop the first-generation PDXs, surgical tumor samples, including lung cancer, gastric carcinoma and hepatoma carcinoma were transplanted subcutaneously into 3 to 6 NSI mice. Tumors that reached an approximate size of 1000 mm^3^ were removed and passed into secondary recipients for expansion for further cancer research. Tumors were cut into 2 × 2 × 2 mm^3^pieces and transplanted into the right flanks of NSI mice. Then, 10, 15, 20 or 24 days after tumor transplantation, mice were infused with CAR T cell or control T cells. In total, 5 × 10^6^ CAR T cells were injected one time into each mouse. Tumors were measured with a caliper, and tumor volume was calculated using the following equation: (length×width×width)/2.

## Results

### dPD1z T and CARPD-L1z T cells efficiently lysed PD-L1^+^ tumor cells

**Fig. 1:**
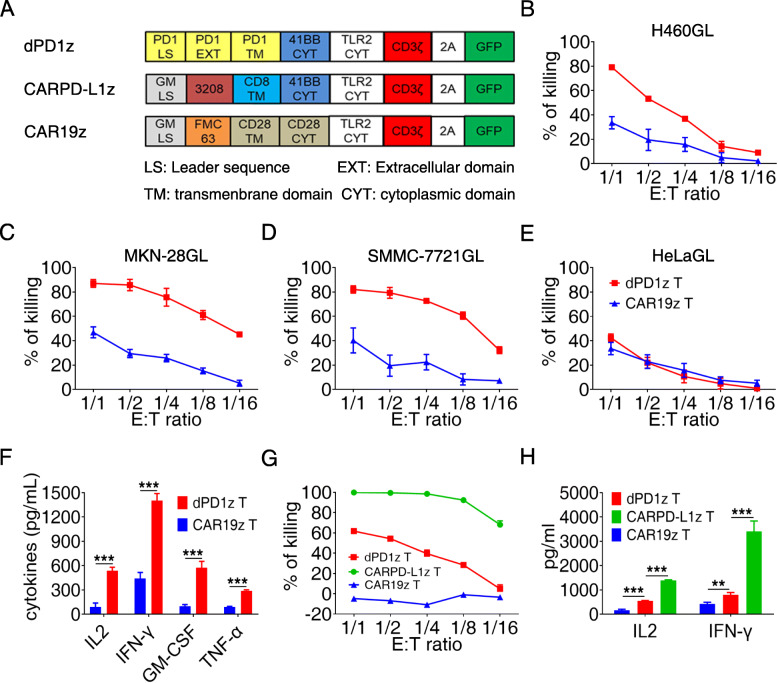
dPD1z T and CARPD-L1z T cells efficiently lysed PD-L1^+^ cancer cells. **a** Schematic diagram of the CAR construction of dPD1z, CARPD-L1z, and CAR19z. **b**-**e** In vitro killing of dPD1z T cells against PD-L1^+^ tumor cells. dPD1z T and CAR19z T were co-cultured with four different types of human cancer cell lines at the indicated effector to target (E: T) ratios for 24 h, and the luciferase activities were calculated to determine the percentages of cytolysis. **f** The production of IL-2, IFN-γ, GM-CSF, and TNF-α after dPD1z T or CAR19z T cells co-culture with H460GL cell line for 24 h at a definitive E: T ratio (1: 1). Error bars denote SD, and the results were compared by unpaired t-test. *** *P* < 0.001. **g** In vitro killing of CARPD-L1z T and dPD1z T cells against H460GL cell line. Each type of cell was co-cultured with H460GL cell line at the indicated effector to target (E: T) ratios for 24 h, and the luciferase activities were calculated to determine the percentages of cytolysis. **h** The production of IL-2 and IFN-γ of CARPD-L1z T, dPD1z T or CAR19z T cells post co-cultured with H460GL cell line for 24 h at a definitive E: T ratio (1: 1). Error bars denote SD, and the results were compared by unpaired t-test. ** *P* < 0.01, and *** *P* < 0.001

To redirect T cells attack tumor cells expressing PD-L1, we designed a dominant-negative form of PD-1, dPD1z, a vector containing the extracellular and transmembrane regions of human PD-1, and a CAR vector against PD-L1, CARPD-L1z, a vector with a high-affinity scFv against human PD-L1. These two vectors share the same intracellular structure, including 4-1BB and TLR2 co-stimulatory domains [40, 41], and the CD3ζ signaling domain, tagged with a green fluorescent protein (GFP) to facilitate the identification of transduced cells (Fig. 1a). The transduction efficiency of these CAR T cells was detected following lentiviral infection (Figure S1A).

To evaluate the functions of these two types of T cells, we performed in vitro functional assays, and found that dPD1z T cells exhibited potent cytotoxicity against H460GL, MKN-28GL, and SMMC-7721GL cells, three of which are PD-L1 positive, but not for HeLaGL cells (Fig. 1b-e), in which PD-L1 expression is much lower (Figure S1C-E). In addition, dPD1z T cells secreted significantly more IL-2, IFN-γ, GM-CSF, and TNF-α once co-cultured with PD-L1^+^ H460GL cells, compared with control CAR19z T cells (Fig. 1f). Of interest, the cytotoxicity of CARPD-L1z T cells was significantly higher than that of dPD1z T cells (Fig. 1g), possibly due to higher affinity of scFv in CARPD-L1z against PD-L1, compared with natural PD-1. As expected, the amounts of IL2 and IFN-γ secreted by CARPD-L1z T cells were significantly higher than those from dPD1z T cells (Fig. 1h). Thus, both dPD1z T and CARPD-L1z T cells are capable of specifically recognizing and lysing PD-L1^+^ tumor cells and secreting cytokines in vitro.

### dPD1z T and CARPD-L1z T cells inhibited the growth of multiple types of tumors in vivo

**Fig. 2:**
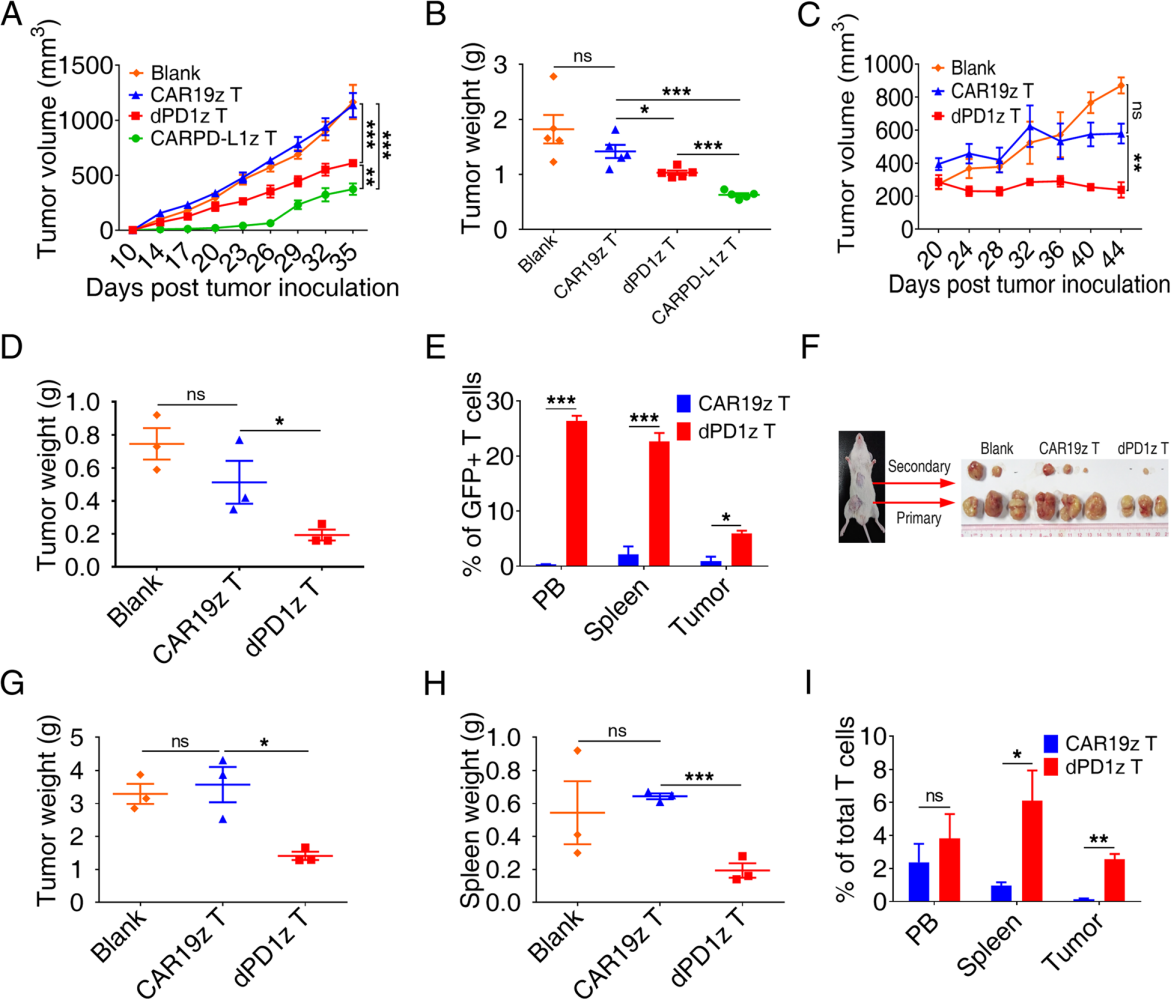
dPD1z T and CARPD-L1z T inhibited the growth of multiple types of PD-L1^+^ tumors in vivo. **a** Tumor volumes and **b** tumor weights of H460GL cells xenograft mice after treatment with CARPD-L1z T, dPD1z T, CAR19z T cells or untreated controls (Blank). NSI mice were transplanted with H460GL cells (5 × 10^5^) at day 0, subsequently, CARPD-L1z T, dPD1z T or CAR19z T (5 × 10^6^) cells were infused at day 10. Tumor volumes were monitored at indicated days and tumor weights were measured after mice euthanasia. The result of tumor volume represent mean ± SEM, and was compared by two-way ANOVA with Tukey’s multiple comparisons test. ** *P* < 0.01, and *** *P* < 0.001. The result of tumor weight represent mean ± SD, and was compared by unpaired t-test. * *P* < 0.05, ** *P* < 0.01, and *** *P* < 0.001. **c** Tumor volumes and **d** tumor weights of NSCLC PDX mice after treatment with dPD1z T, CAR19z T cells or untreated controls (Blank). NSI mice were transplanted with primary NSCLC cells at day 0, subsequently, dPD1z T or CAR19z T (5 × 10^6^) cells were infused twice at day 15 and day 20. Tumor volumes were monitored at indicated days and tumor weights were measured after mice euthanasia. The result of tumor volume represent mean ± SEM, and was compared by two-way ANOVA with Tukey’s multiple comparisons test. ** *P* < 0.01. The result of tumor weight represent mean ± SD, and was compared by unpaired t-test. * *P* < 0.05. **e** Percentages of GFP^+^ T cells in peripheral blood (PB), spleen, and tumors after treated with dPD1z T or CAR19z T cells (gated on live cells) are shown. Error bars denote SD, and the results were compared by unpaired t-test. * *P* < 0.05, ** *P* < 0.01, and *** *P* < 0.001. **f** Primary and secondary tumor images of gastric cancer PDX models after treatment with dPD1z T, CAR19z T cells or untreated controls (blank). **g** Primary tumor weights and **h** spleen weights of gastric cancer PDX after treatment with dPD1z T, CAR19z T cells or untreated controls (Blank). NSI mice were transplanted with primary gastric cancer cells at day 0, subsequently, dPD1z T or CAR19z T (5 × 10^6^) cells were infused twice at day 20 and day 24. Tumor weights and spleen weights were measured at day 45 after mice euthanasia. The results represent mean ± SD, and were compared by unpaired t-test. * *P* < 0.05, ** *P* < 0.01, and *** *P* < 0.001. **i** Percentages of T cells in peripheral blood (PB), spleen, and tumors after treated with dPD1z T or CAR19z T cells (gated on live cells) are shown. Error bars denote SD, and the results were compared by unpaired t-test. * *P* < 0.05, and ** *P* < 0.01

The anti-tumor efficacy of dPD1z T and CARPD-L1z T cells were subsequently examined in a cell line-derived xenografts (CDX). Consistent with results of in vitro cytotoxicity assays, both dPD1z T and CARPD-L1z T cells inhibited the growth of H460GL cells in immunodeficient NSI (NOD/SCID/IL-2 g^−/−^) mice [42, 43], and CARPD-L1z T cells showed superior anti-tumor effects (Fig. 2a-b).

We next evaluated the tumor-suppressive capacities of dPD1z T cells in several PD-L1^+^ personal derived xenografts (PDX). We first confirmed the anti-tumor activity of dPD1z T cells in a non-small cell lung cancer (NSCLC) PDX (P1) (Figure S2A-B) [44]. Remarkably, tumors in the dPD1z T-cell group stopped growth after twice infusions of dPD1z T cells, whereas tumors in the CAR19z T-cell group grew robustly (Fig. 2c-d). Consistent with this observation, the percentages of GFP^+^ T cells in the peripheral blood (PB), spleen, and tumors of the dPD1z T-cell group were significantly higher than those of CAR19z T-cell group (Fig. 2e).

We found that dPD1z T cells not only repressed the growth of subcutaneous tumors but also impeded cancer cell metastasis in metastatic gastric cancer (P2) PDX (Figure S2A-B). Both primary and secondary tumors in dPD1z T-cell group were smaller than those of the control groups (Fig. 2f-g). HE staining suggested that tumor cells invaded into the spleens (Figure S3A) and caused splenomegaly (Figure S3B). In contrast, the spleens of the dPD1z T-cell group were smaller (Fig. 2h and S3B) compared with those of the control groups. Consistently, the T-cell percentage in spleens and tumors of dPD1z T-cell group were greater than those in the control groups (Fig. 2i). Similar to the NSCLC and gastric PDXs, dPD1z T cells also inhibited tumor growth in a hepatoma carcinoma PDX (P3) (Figures S2A-B and S3C-D). Taken together, dPD1z T cells suppressed tumor growth and metastasis in multiple cancer PDXs and elevated the percentages of tumor-infiltrating lymphocytes.

### Combined CARMSLNz T with CARPD-L1z T or dPD1z T cells failed to achieve a synergistic anti-tumor effect in vivo

**Fig. 3:**
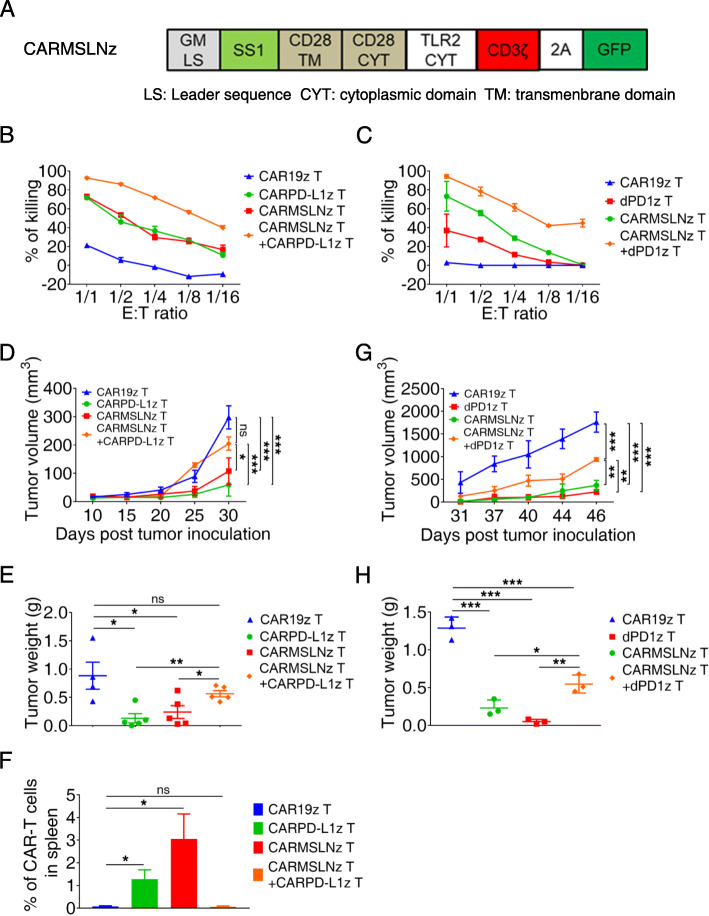
Combined CARMSLNz T with CARPD-L1z T or dPD1z T cells failed to achieve a synergistic anti-tumor effect in vivo. **a** Schematic diagram of the CAR construction of CARMSLNz. **b** In vitro killing of CARPD-L1z T, CARMSLNz T, the combination of CARMSLNz T and CARPD-L1z T and CAR19z T cells against H460-MSLNGL cell line. Each type of cell was co-cultured with H460-MSLNGL cell line at the indicated effector to target (E: T) ratios for 24 h, and the luciferase activities were calculated to determine the percentages of cytolysis. **c** In vitro killing of dPD1z T, CARMSLNz T, the combination of CARMSLNz T and dPD1z T and CAR19z T cells against H460-MSLNGL cell line. Each type of cell was co-cultured with H460-MSLNGL cell line at the indicated effector to target (E: T) ratios for 24 h, and the luciferase activities were calculated to determine the percentages of cytolysis. **d** Tumor volumes and **e** tumor weights of NSCLC PDX (P4) after treatment with CARPD-L1z T, CARMSLNz T, the combination of CARMSLNz T and CARPD-L1z T or CAR19z T cells. NSI mice were transplanted with primary NSCLC cells at day 0, subsequently, CAR T (5 × 10^6^) cells were infused on day 10. Tumor volumes were monitored at indicated days and tumor weights were measured after mice euthanasia. The result of tumor volume represent mean ± SEM, and was compared by two-way ANOVA with Tukey’s multiple comparisons test. * *P* < 0.05, ** *P* < 0.01, and *** *P* < 0.001. The result of tumor weight represent mean ± SD, and was compared by unpaired t-test. * *P* < 0.05, and ** *P* < 0.01. **f** Percentages of CAR T cells in the spleen after treated with each type of CAR T cells (gated on live cells) are shown. Error bars denote SD, and the results were compared by unpaired t-test. * *P* < 0.05. **g** Tumor volumes and **h** tumor weights of NSCLC PDX (P4) after treatment with dPD1z T, CARMSLNz T, the combination of CARMSLNz T and dPD1z T or CAR19z T cells. NSI mice were transplanted with primary NSCLC cells at day 0, subsequently, CAR T (5 × 10^6^) cells were infused twice at day 10 and day 20. Tumor volumes were monitored at indicated days and tumor weights were measured after mice euthanasia. The result of tumor volume represent mean ± SEM, and was compared by two-way ANOVA with Tukey’s multiple comparisons test. ** *P* < 0.01, and *** *P* < 0.001. The result of tumor weight represent mean ± SD, and was compared by unpaired t-test. * *P* < 0.05, ** *P* < 0.01, and *** *P* < 0.001

It has been reported that PD-L1 expression by tumor cells can inhibit the anti-tumor activities of CAR-T cells targeting tumor-specific antigens (TSAs) [45], so we hypothesized that elimination of PD-L1 expressing tumor cells by CARPD-L1z T or dPD1z T cells will show synergistic activities with traditional CAR-T cells targeting TSAs. To test our hypothesis, we firstly mixed CARPD-L1z T cells and anti-mesothelin CAR T cells (CARMSLNz T cells) (Fig. 3a) with 1: 1 ratio and co-cultured them with H460-MSLNGL that expressed Mesothelin (Figure S4A), an antigen widely expressed in lung cancer and gastric cancers [46, 47]. Remarkably, the combination of CARPD-L1z T and CARMSLNz T cells showed the more potent lysing capacity than individual CARPD-L1z T or CARMSLNz T cells, though the total CAR T cell numbers were equivalent (Fig. 3b). IL2 and IFN-γ secretion was detected in all three co-cultures but not in the co-culture with CAR19z T cells (Figure S4B-C). Similarly, the cytotoxicity of dPD1z T and CARMSLNz T cells in the combination group was the highest (Fig. 3c).

To further test our hypothesis in vivo, we next transplanted tumor cells from an NSCLC patient sample (P4) that highly expressed both PD-L1 and Mesothelin in NSI mice (Figure S2C), followed by injection of equivalent numbers of CARMSLNz T, CARPD-L1z T, and a combination of CARMSLNz T and CARPD-L1z T cells, or CAR19z T cells with similar transduction efficiencies (Figure S1B). Compared with CAR19z T cells, both CARMSLNz T and CARPD-L1z T cells individually inhibited tumor progression in xenografts (Fig. 3d). To our surprise, tumors in the combination group were significantly larger and heavier than that in the CARMSLNz T or CARPD-L1z T-cell groups (Fig. 3d-e). In addition, much fewer CARMSLNz or CARPD-L1z T cells were detected in the spleen from the combination group (Fig. 3f and S5). Similarly, suppression of tumor growth in the combination group of CARMSLNz T and dPD1z T cells is not as efficient as the individual treatment of CARMSLNz T or dPD1z T cells (Fig. 3g-h). Therefore, combining CARMSLNz T with CARPD-L1z T or dPD1z T cells did not achieve a significant synergistic anti-tumor effect in vivo.

### CARPD-L1z T cells lysed PD-L1^+^ T cells

**Fig. 4:**
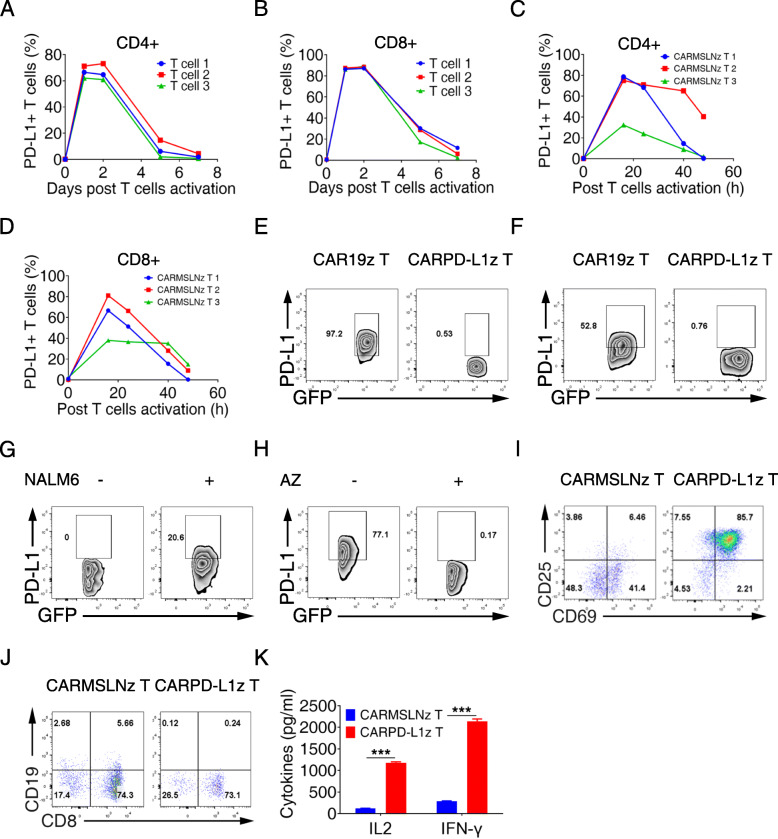
CARPD-L1z T cells lysed PD-L1^+^ T cells. Percentage of PD-L1^+^ T cells in **a** CD4^+^ T cells (gated on CD3^+^CD8^−^ cells) and **b** CD8^+^ T cells (gated on CD3^+^CD8^+^ cells) post activated by CD3 and CD28 antibodies. FACS detection of PD-L1 expression at indicated time points. Percentage of PD-L1^+^ T cells in **c** CD4^+^ CARMSLNz T cells (gated on CD3^+^GFP^+^CD4^+^ cells) and **d** CD8^+^ CARMSLNz T cells (gated on CD3^+^GFP^+^CD8^+^ cells) post co-cultured with H460-MSLNGL cells. CARMSLNz T cells were co-cultured with H460-MSLNGL cells for 0 h, 16 h, 24 h, 40 h and 48 h at a definitive E: T ratio (1: 1), then the expression of PD-L1 was detected by FACS. **e** Percentage of PD-L1^+^ T cells in CAR19z T and CARPD-L1z T cells (gated on CD3^+^GFP^+^ cells) post activated by CD3 and CD28 antibodies for 16 h. **f** Percentage of PD-L1^+^ T cells in CAR19z T cells (gated on CD3^+^GFP^+^ cells) post co-cultured with NALM6 cells for 24 h at a definitive E: T ratio (2: 1), and percentage of PD-L1^+^ T cells in CARPD-L1z T cells (gated on CD3^+^GFP^+^ cells) post co-cultured with H460GL cells for 24 h at a definitive E: T ratio (2: 1). **g** Percentage of PD-L1^+^ T cells in CAR19BBz T cells (gated on CD3^+^GFP^+^ cells) post co-cultured with or without NALM6 cells. CAR19BBz T cells were co-cultured with or without NALM6 cells for 12 h, at a definitive E: T ratio (2: 1), then the expression of PD-L1 was detected by FACS. **h** Percentage of PD-L1^+^ T cells in CARMSLNz T cells (gated on CD3^+^GFP^+^ cells) post co-cultured with H460-MSLNGL cells with or without AZ (20 μg/mL) for 24 h at a definitive E: T ratio (1: 1). **i** Percentage of CD25^+^CD69^+^ T cells in CARPD-L1z T and CARMSLNz T cell (gated on GFP^+^ cells) post co-cultured with uPD-L1 T cells at a definitive E: T ratio (1: 1) for 24 h. **j** Percentage of CD19^+^ T cells (uPD-L1 T cells) after co-cultured with CARPD-L1z T or CARMSLNz T cell at a definitive E: T ratio (1: 1) for 24 h (gated on live cells). **k** The production of IL-2 and IFN-γ of CARPD-L1z T or CARMSLNz T cells post co-culture with uPD-L1 T cells for 24 h at a definitive E: T ratio (1: 1). Error bars denote SD, and the results were compared by unpaired t-test. * *P* < 0.05, ** *P* < 0.01, and *** *P* < 0.001

Previous studies suggested that PD-L1 is expressed not only in tumor cells but also in activated T cells [48, 49]. Indeed, we found that both CD4^+^ T (Fig. 4a and S6A) and CD8^+^ T cells (Fig. 4b and S6B) up-regulated PD-L1 expression upon CD3 and CD28 antibodies activation within 1 day. Moreover, both CD4^+^ (Fig. 4c and S7A) and CD8^+^ (Fig. 4d and S7B) CARMSLNz T cells started to express PD-L1 and kept its expression for 40 h after co-cultured with H460-MSLNGL tumor cells. Surprisingly, we didn’t observe any PD-L1 up-regulation of CARPD-L1z T cells either activated by CD3/CD28 antibodies or co-cultured with PD-L1^+^ tumor cells (Fig. 4e-f), even the percentage of CD25 and CD69 double-positive T cells confirmed they fully activated (Figure S8A-B). Both CAR19z and CARMSLNz adopted CD28 co-stimulatory molecules, but CARPD-L1z contained 4-1BB co-stimulatory molecules. To exclude the possibility that different co-stimulatory molecules result in different PD-L1 expression on CAR-T cells after activation, we constructed CAR19BBz, whose structure is same as CAR19z expect for 4-1BB co-stimulatory domain. We found that the PD-L1 expression of CAR19BBz T cells was up-regulated after co-culturing with NALM6 cells (Fig. 4g). The scFv of CARPD-L1z is derived from anti-PD-L1 antibody AZ. Interestingly, when we added AZ into the co-culture system of CARMSLNz T cells with H460-MSLNGL tumor cells, we failed to detect PD-L1 expression on CARMSLNz T cells (Fig. 4h). We hypothesized that CARMSLNz T cells were killed by CARPD-L1z T in xenografts when CARMSLNz T cells up-regulated PD-L1 expression upon activation with tumor cells, but CARPD-L1z T cells didn’t kill each other. Indeed, when we co-cultured T cells that overexpressed PD-L1 (uPD-L1 T) (Figure S8C-E) with CARPD-L1z T, or CARMSLNz T cells as negative control, we found that CARPD-L1z T cells up-regulated CD25 and CD69 expression, suggesting that they were activated by PD-L1 overexpressing T cells (Fig. 4i). In addition, percentages of PD-L1 expressing T cells tagged with tCD19 decreased (Fig. 4j) and large amounts of IL-2 and IFN-γ were detected (Fig. 4k) in the co-culture with CARPD-L1z T cells. In summary, these results suggest that CARPD-L1z T cells killed CARMSLNz T cells that upregulated PD-L1 expression upon activation by tumor cells in xenografts, this may be the reason why we failed to achieve a synergistic anti-tumor effect when combining CARMSLNz T with CARPD-L1z T or dPD1z T cells.

## Discussion

The expression of PD-L1, which serves as a ligand for PD1 on T cells to protect tumor cells from immune control mediated by T cells, is elevated in many types of solid tumors [50]. Compared with anti-PD1 and anti-PD-L1 antibodies, several groups have reported the modification of traditional CAR-T cells with a PD1 switching receptor containing a CD28 intracellular domain [37–39]. These CAR-T cells convert PD-L1 inhibitory signals into CD28 co-stimulatory signals, which protect CAR T cells from PD-L1 mediated suppression. However, these CAR T cells have to rely on the recognition of TSAs by traditional CARs due to the lack of CD3 domain in their PD-1 switching receptor, limiting their efficacy against heterogeneous tumors. Here, dPD1z T and CARPD-L1z T cells not only switch inhibitory signals into activating signals upon encountering PD-L1 ligands by their CAR molecules, which contain both CD3ζ and co-stimulatory domains, but also are capable of eliminating PD-L1-expressing tumor cells.

The dPD1z was a dominant-negative form of PD-1, its extracellular and transmembrane regions is derived from natural PD-1, so dPD1z T cells can target PD-L1 and PD-L2 simultaneously. But the binding affinity with PD-L1 and PD-L2 of dPD1z is limited. In order to improve the safety and binding affinity of anti-PD-L1 CAR-T cells, we design CARPD-L1z, which contain a high-affinity scFv against human PD-L1, and can only target PD-L1. The in vitro killing assay suggested that the cytotoxicity of CARPD-L1z T cells was significantly higher than that of dPD1z T cells, and the amounts of IL2 and IFN-γ secreted by CARPD-L1z T cells were significantly higher than those from dPD1z T cells.

Besides tumor cells, PD-L1 is also expressed in various types of cells, including activated T cells, NK cells, dendritic cells (DC) and myeloid-derived suppressor cells (MDSCs) [49, 51–54]. In this study, we preliminarily verified the feasibility and efficacy of CAR-T cells targeting PD-L1 for the treatment of solid tumors, and we found that PD-L1 was up-regulated in T cells that were activated through either endogenous TCR or CAR signaling, making these activated T cells be the target of anti-PD-L1 T cells. This explains why a combination of CARMSLNz T with dPD1z T or CARPD-L1z T cells did not achieve synergistic anti-tumor effects in PDX. In the future work, we will improve and optimize these CAR-T cells to reduce their potential off-target toxicity, for example, by combining anti-PD-L1 CAR-T cells with SynNotch system [55]. In this sense, only when another CAR is activated by its cognate tumor antigen, anti-PD-L1 CARs will be expressed, and then activated.

Despite we detected PD-L1 expression on activated T cell or CAR-T cells, however, we failed to observed PD-L1 expression on CARPD-L1z T cells no matter being activated by CD3 and CD28 antibodies or PD-L1^+^ tumor cells. In order to exclude the influence of co-stimulatory molecules on the expression level of PD-L1, we detected the PD-L1 expression of CAR19BBz T cells which containing FMC63 scFv, 4-1BB and TLR2 co-stimulatory molecules, and the result showed that 4-1BB co-stimulatory molecule did not affect the expression of PD-L1. We consider that the low expression of PD-L1 on CARPD-L1z T cells was due to the binding of anti-PD-L1 scFv with PD-L1 on the membrane of individual cells, and then caused PD-L1 endocytosis. This was supported by the fact that when we add anti-PD-L1 antibody AZ into the co-culture system of CARMSLNz T cells with H460-MSLNGL tumor cells, we failed to detect PD-L1 expression on CARMSLNz T cells. The scFv of CARPD-L1z is derived from AZ, further and deeper reason need us to be carried out.

## Conclusions

In conclusion, we demonstrate that dPD1z T and CARPD-L1z T cells were effective for suppressing the growth of multiple types of PD-L1^+^ solid tumors in PDX models and the combination of CARMSLNz T with dPD1z T or CARPD-L1z T cells did not show synergistic efficacy. Since PD-L1 is expressed in some normal tissues, we need to fully evaluate the safety of dPD1z T and CARPD-L1z T cells in animal models before conducting a clinical trial for treating solid tumors.

## Supplementary information

**Additional file 1: Supplemental Figure 1.** Transduction efficiency of CAR-T cells and GFP and PD-L1 expression of GL transduced cancer cell lines. (A) Transduction efficiency of CAR-T cells used in in vitro cytotoxicity assays. (B) Transduction efficiency of CAR-T cells used to evaluate the anti-tumor efficacy of the combination of CARMSLNz T and dPD1z T and the combination of CARMSLNz T and CARPD-L1z T cells in NSCLC (P4) PDX. (C) Schematic diagram of the GL vector. FACS analysis of (D) GFP and (E) PD-L1 expression levels in multiple cancer cell lines transduced with GL. **Supplemental Figure 2.** PD-L1 expression in primary tumor samples. (A) qRT-PCR and (B) FACS analysis PD-L1 expression in primary NSCLC cells (P1), gastric cancer cells (P2) and hepatoma carcinoma cells (P3). (C) The expression of PD-L1 and Mesothelin (MSLN) in primary NSCLC (P4) cells. **Supplemental Figure 3.** dPD1z T cells inhibit tumor growth in gastric cancer and hepatoma carcinoma PDXs. (A) IHC images of a normal spleen (left) and a spleen with metastatic tumors (right). (B) Images of spleens from gastric cancer PDXs after treatment with dPD1z T, CAR19z T or untreated controls (blank). (C) Tumor volumes and (D) tumor weights of hepatoma carcinoma PDXs (P3) after treatment with dPD1z T, CAR19z T cells or untreated controls (Blank). NSI mice were transplanted with hepatoma carcinoma cells at day 0, subsequently, dPD1z T or CAR19z T (5 × 10^6^) cells were infused twice at day 15 and day 20. Tumor volumes were monitored at indicated days and tumor weights were measured after mice euthanasia. The result of tumor volume represent mean ± SEM, and was compared by two-way ANOVA with Tukey’s multiple comparisons test. * *P* < 0.05. The result of tumor weight represent mean ± SD, and was compared by unpaired t-test. ** *P* < 0.01. **Supplemental Figure 4.** The production of IL-2 and IFN-γ of CARMSLNz T, CARPD-L1z T, the combination of CARMSLNz T and CARPD-L1z T or CAR19z T cells post co-cultured with H460-MSLNGL cells. (A) FACS detection of Mesothelin (MSLN) expression of H460GL and H460-MSLNGL cells. The production of (B) IL-2 and (C) IFN-γ after CARMSLNz T, CARPD-L1z T, the combination of CARMSLNz T and CARPD-L1z T or CAR19z T cells co-cultured with H460-MSLNGL cell line for 24 h at a definitive E: T ratio (1: 1). Error bars denote SD, and the results were compared by unpaired t-test. * *P* < 0.05, ** *P* < 0.01, and *** *P* < 0.001. **Supplemental Figure 5.** Percentages of CAR T cells in the spleen of NSCLC PDXs (P4) after treated with CARMSLNz T, CARPD-L1z T, the combination of CARMSLNz T and CARPD-L1z T or CAR19z T cells (gated on live cells). **Supplemental Figure 6.** The expression of PD-L1 in the activated T cells. Percentage of PD-L1^+^ T cells in (A) CD4^+^ T cells (gated on CD3^+^CD8^−^ cells) and (B) CD8^+^ T cells (gated on CD3^+^CD8^+^ cells) post activated by CD3 and CD28 antibodies. FACS detection of PD-L1 expression at indicated time points. **Supplemental Figure 7.** The expression of PD-L1 in CARMSLNz T cells post co-cultured with H460-MSLNGL cells. Percentage of PD-L1^+^ T cells in (A) CD4^+^ CARMSLNz T cells (gated on CD3^+^GFP^+^CD4^+^ cells) and (B) CD8^+^ CARMSLNz T cells (gated on CD3^+^GFP^+^CD8^+^ cells) post co-cultured with H460-MSLNGL cells. CARMSLNz T cells were co-cultured with H460-MSLNGL for 0 h, 16 h, 24 h, 40 h and 48 h at a definitive E: T ratio (1: 1), then the expression of PD-L1 was detected by FACS. **Supplemental Figure 8.** Overexpression PD-L1 in T cells. (A) Percentage of CD25^+^CD69^+^ T cells in CARPD-L1z T and CAR19z T cells (gated on CD3^+^GFP^+^ cells) post activated by CD3 and CD28 antibodies for 16 h. (B) Percentage of CD25^+^CD69^+^ T cells in CAR19z T cells (gated on CD3^+^GFP^+^ cells) post co-cultured with NALM6 cells for 24 h at a definitive E: T ratio (2: 1), and percentage of CD25^+^CD69^+^ T cells in CARPD-L1z T cells (gated on CD3^+^GFP^+^ cells) post co-cultured with H460GL cells for 24 h at a definitive E: T ratio (2, 1). (C) Schematic diagram of uPD-L1 vector. FACS detection of the expression of (D) CD19 and (E) PD-L1 in T cells after transduced with uPD-L1.

## Data Availability

The datasets supporting the conclusions of this article are included within the article and additional files.

## Abbreviations

CAR: Chimeric antigen receptor
MSLN: Mesothelin
scFv: Single-chain fragment variable
TSA: Tumor-specific antigens
NSCLC: Non-small cell lung cancer
PDX: Patient-derived xenograft
GPC3: Glypican 3
