# Supplementary material for: Chimeric antigen receptor T cells targeting PD-L1 suppress tumor growth
Source: Biomark Res. 2020 Jun 3;8:19. doi: 10.1186/s40364-020-00198-0 (PMC7268496; doi:10.1186/s40364-020-00198-0)

Supplemental Figure 1

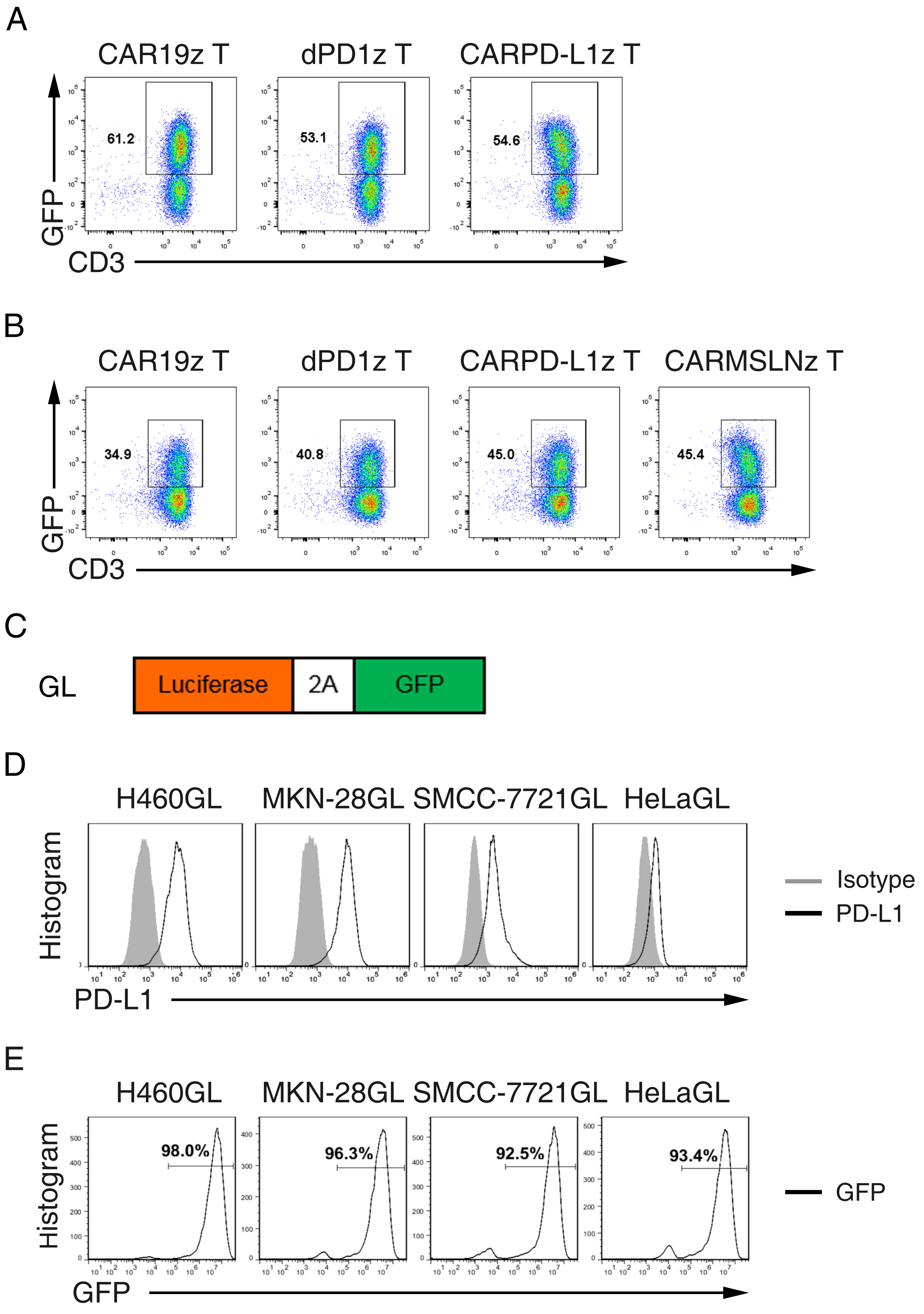

Supplemental Figure 2

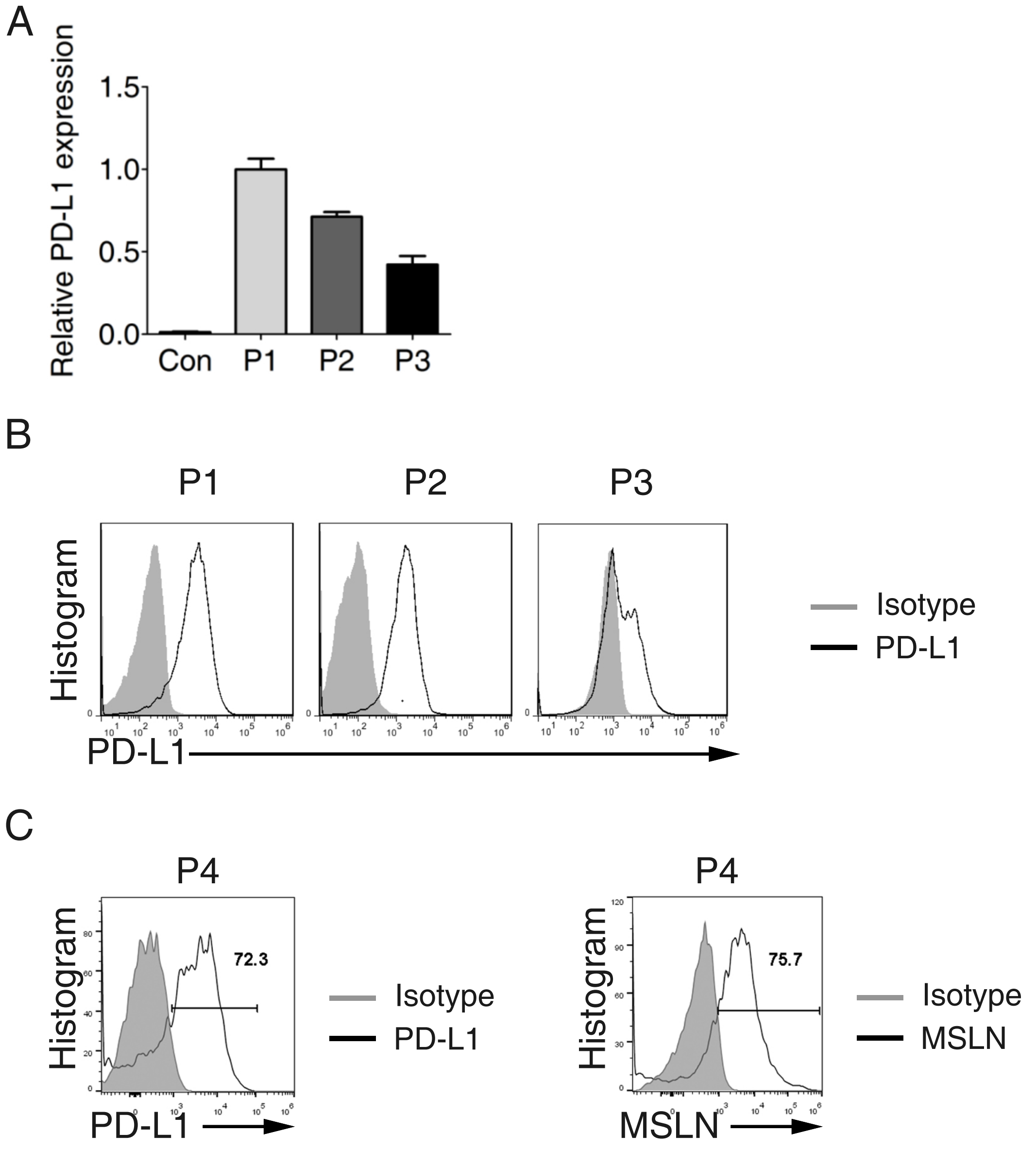

Supplemental Figure 3

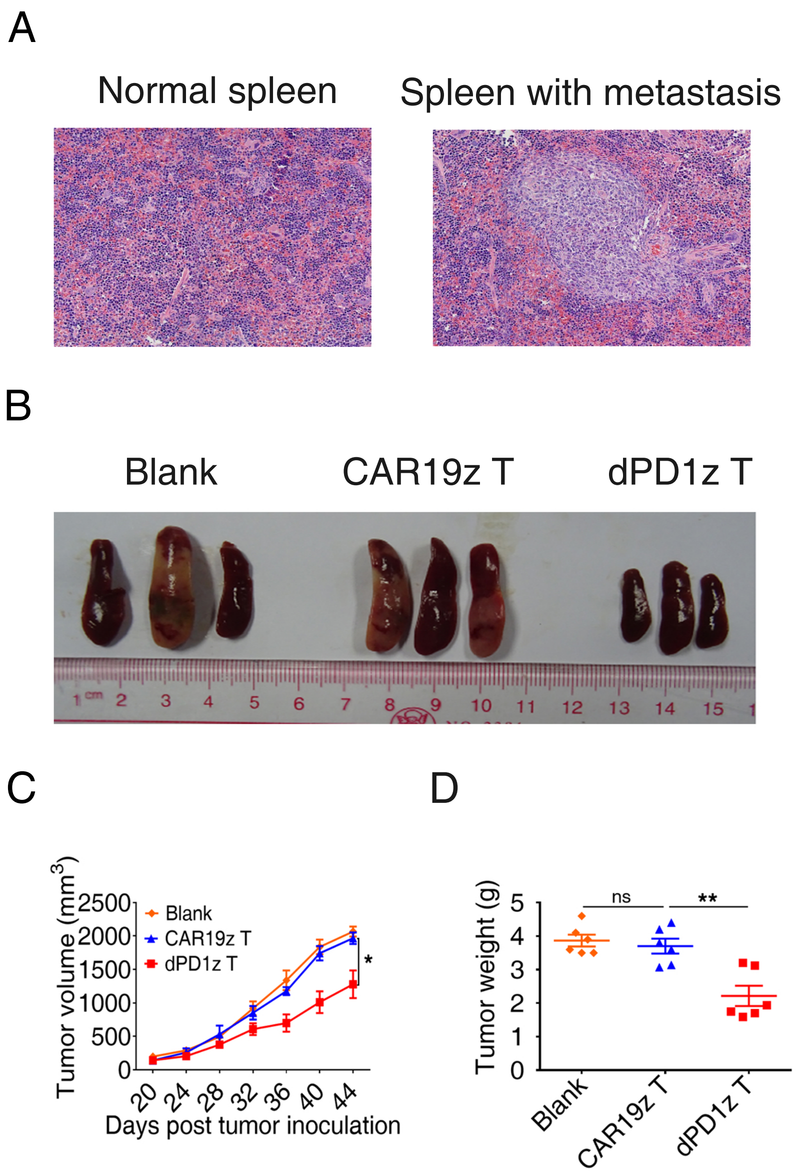

Supplemental Figure 4

A

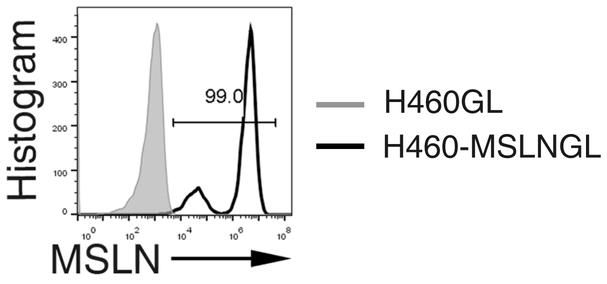

B

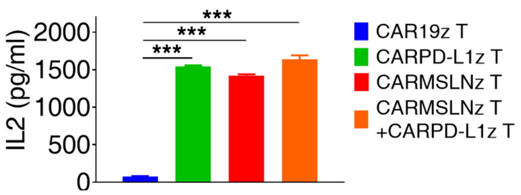

C

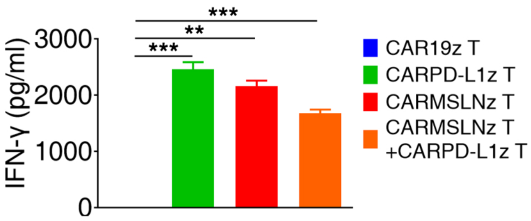

Supplemental Figure 5

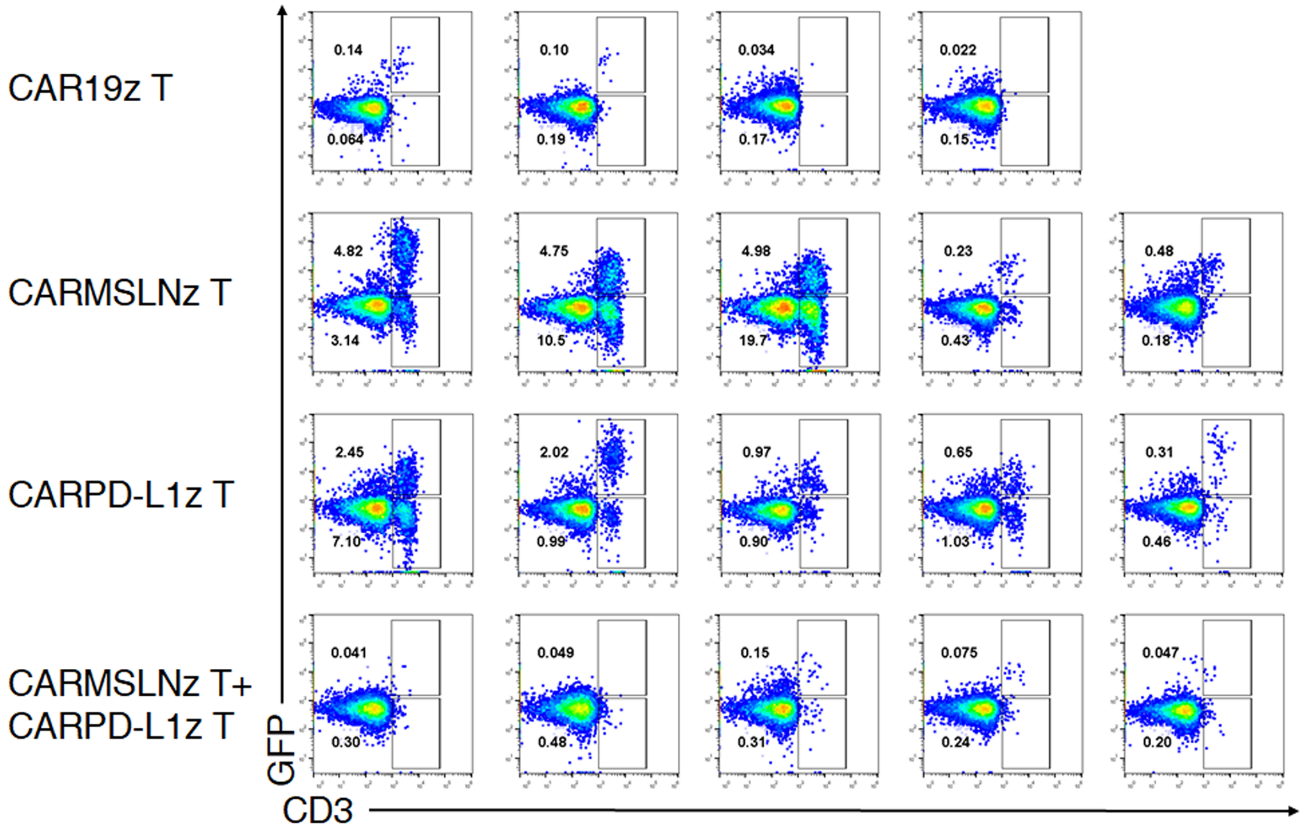

Supplemental Figure 6

A

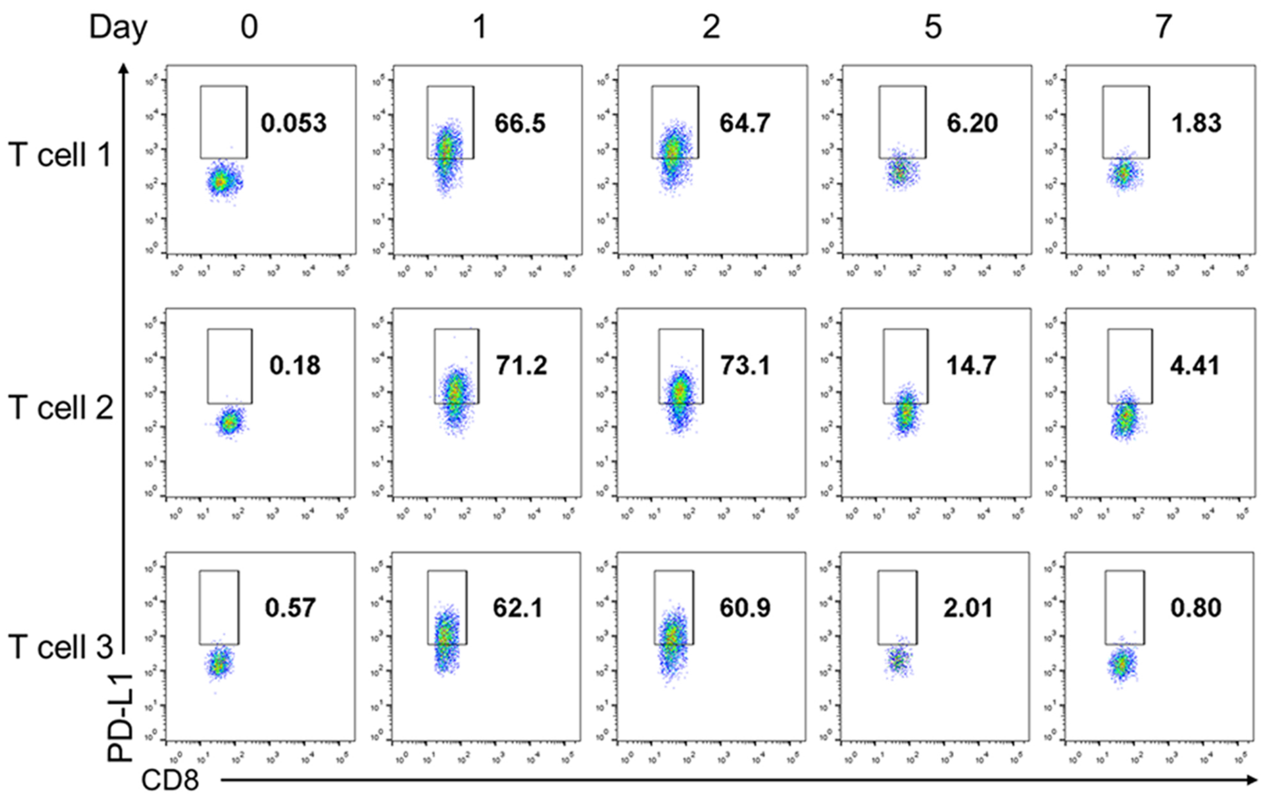

B

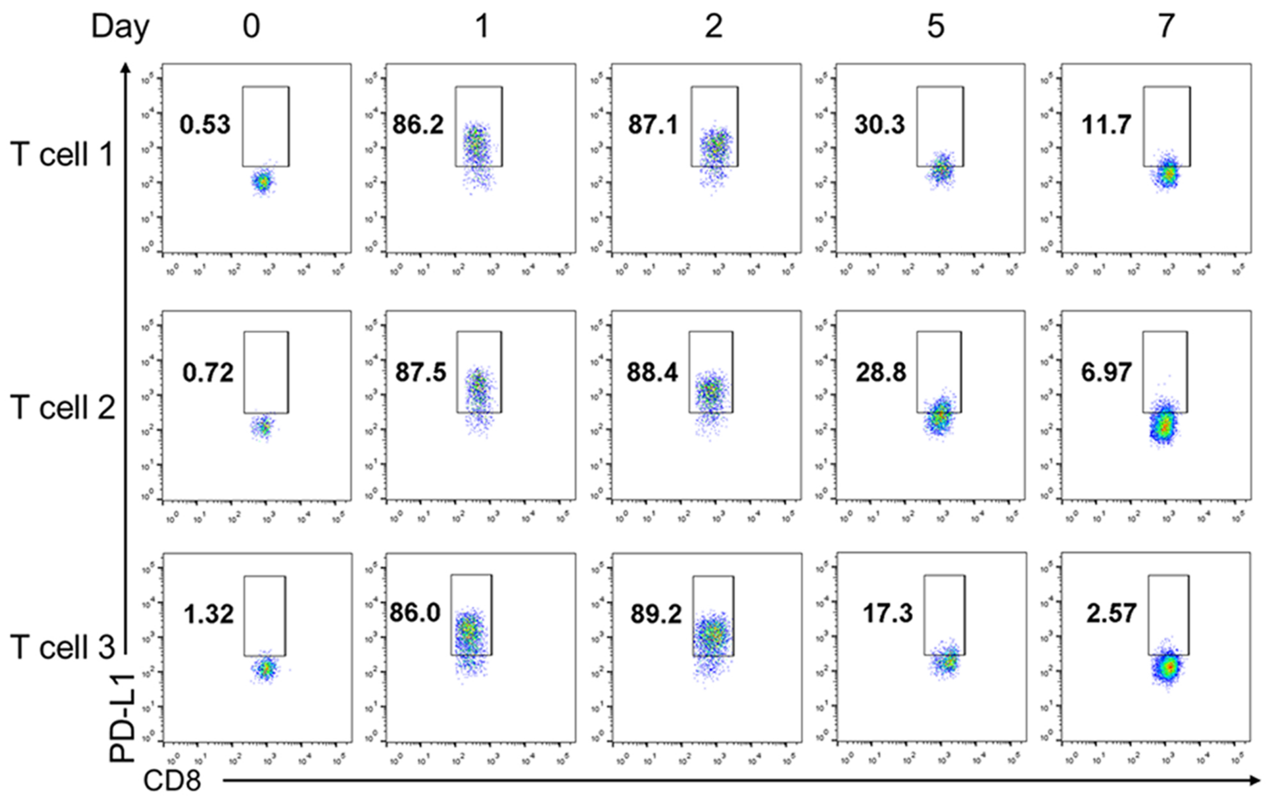

Supplemental Figure 7

A

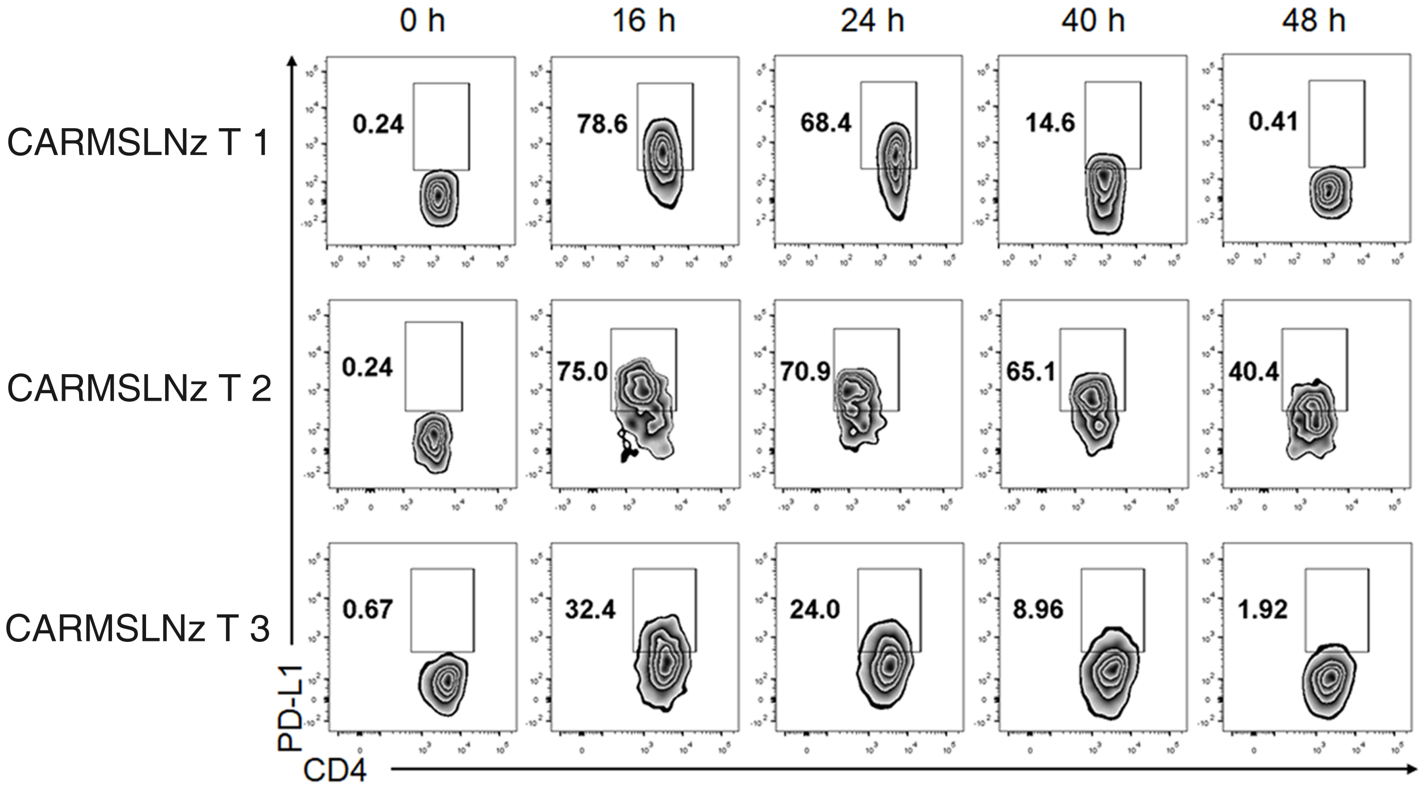

B

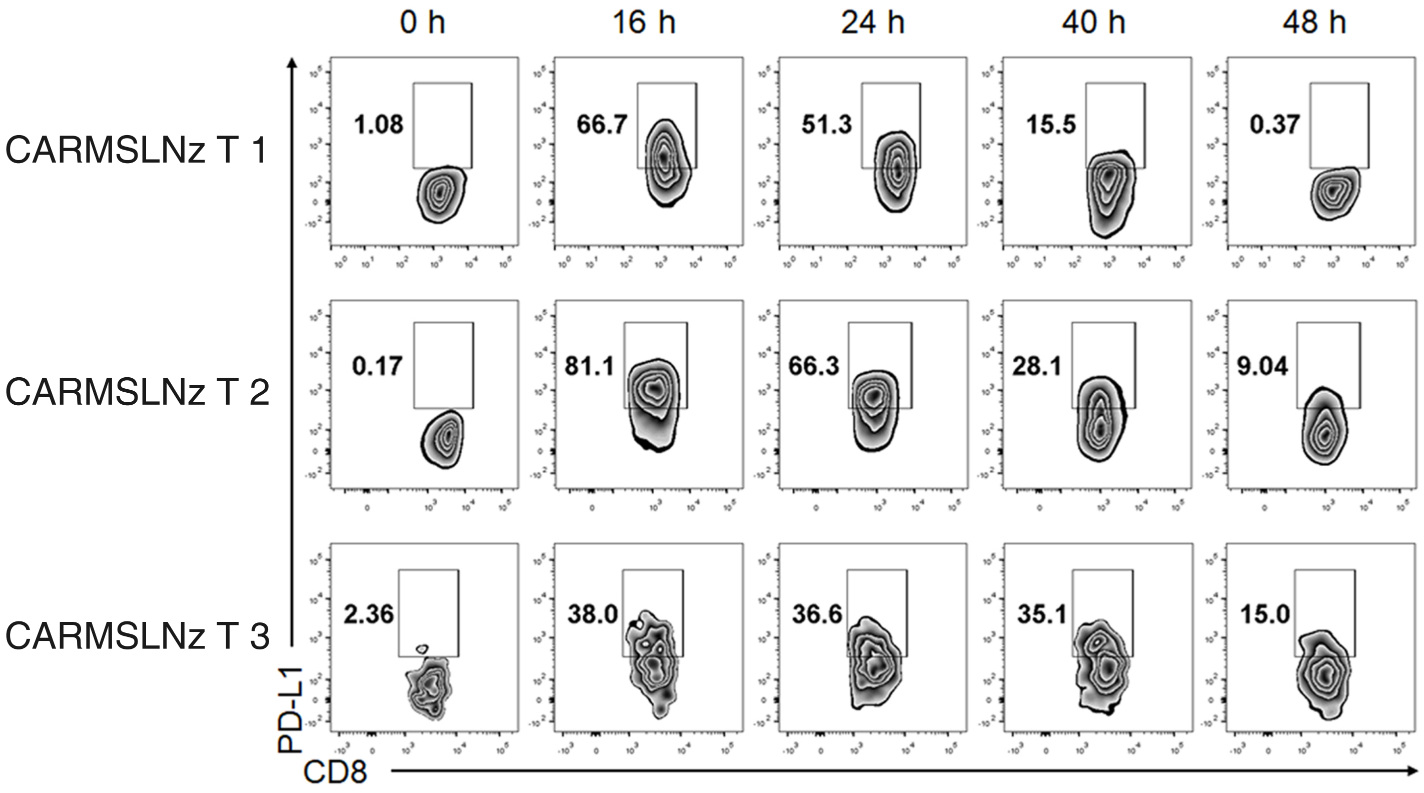

Supplemental Figure 8

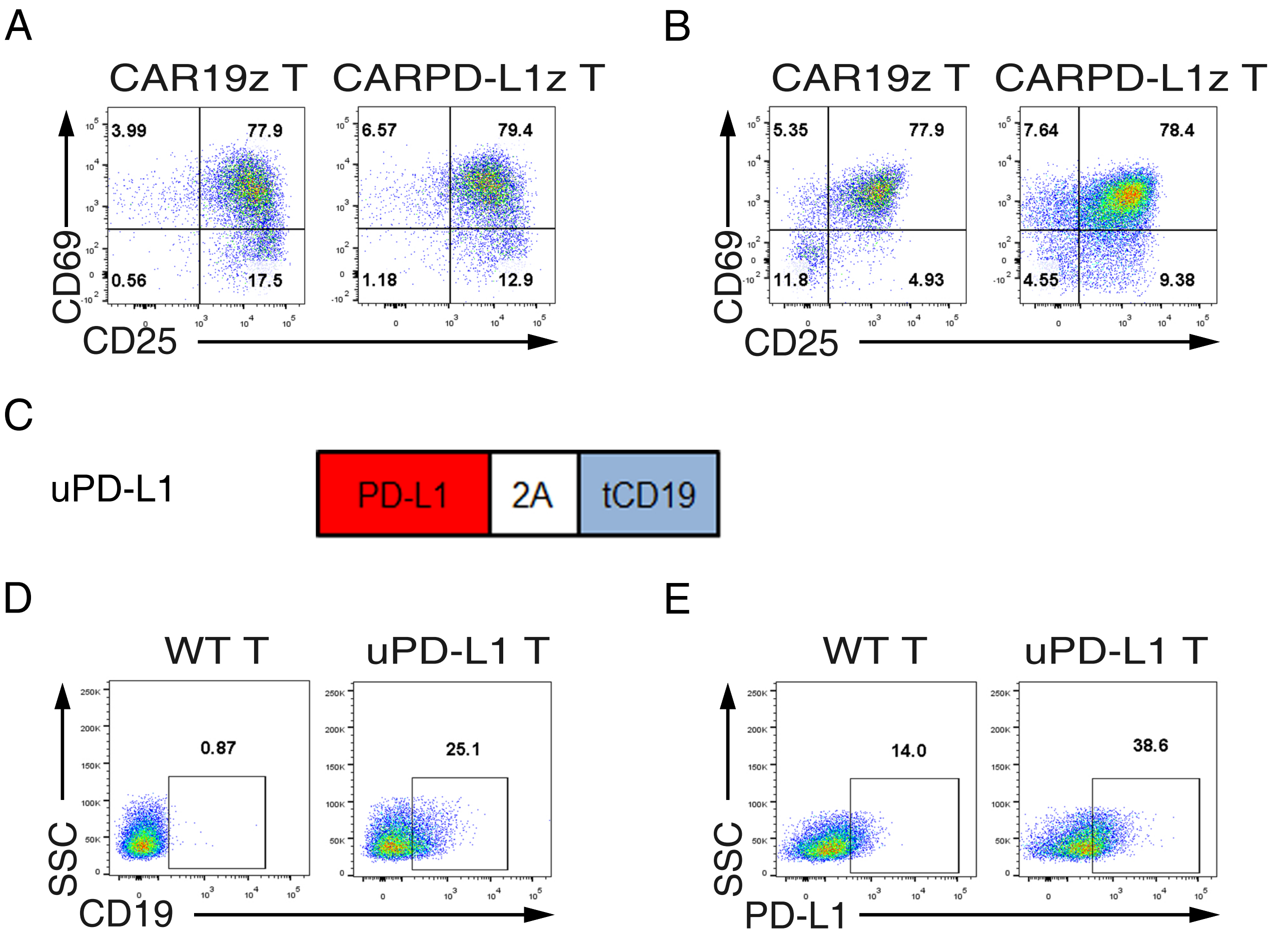

Supplement: Supplementary file 1 — Additional file 1: Supplemental Figure 1. Transduction efficiency of CAR-T cells and GFP and PD-L1 expression of GL transduced cancer cell lines. (A) Transduction efficiency of CAR-T cells used in in vitro cytotoxicity assays. (B) Transduction efficiency of CAR-T cells used to evaluate the anti-tumor efficacy of the combination of CARMSLNz T and dPD1z T and the combination of CARMSLNz T and CARPD-L1z T cells in NSCLC (P4) PDX. (C) Schematic diagram of the GL vector. FACS analysis of (D) GFP and (E) PD-L1 expression levels in multiple cancer cell lines transduced with GL. Supplemental Figure 2. PD-L1 expression in primary tumor samples. (A) qRT-PCR and (B) FACS analysis PD-L1 expression in primary NSCLC cells (P1), gastric cancer cells (P2) and hepatoma carcinoma cells (P3). (C) The expression of PD-L1 and Mesothelin (MSLN) in primary NSCLC (P4) cells. Supplemental Figure 3. dPD1z T cells inhibit tumor growth in gastric cancer and hepatoma carcinoma PDXs. (A) IHC images of a normal spleen (left) and a spleen with metastatic tumors (right). (B) Images of spleens from gastric cancer PDXs after treatment with dPD1z T, CAR19z T or untreated controls (blank). (C) Tumor volumes and (D) tumor weights of hepatoma carcinoma PDXs (P3) after treatment with dPD1z T, CAR19z T cells or untreated controls (Blank). NSI mice were transplanted with hepatoma carcinoma cells at day 0, subsequently, dPD1z T or CAR19z T (5 × 10^6^) cells were infused twice at day 15 and day 20. Tumor volumes were monitored at indicated days and tumor weights were measured after mice euthanasia. The result of tumor volume represent mean ± SEM, and was compared by two-way ANOVA with Tukey’s multiple comparisons test. * P < 0.05. The result of tumor weight represent mean ± SD, and was compared by unpaired t-test. ** P < 0.01. Supplemental Figure 4. The production of IL-2 and IFN-γ of CARMSLNz T, CARPD-L1z T, the combination of CARMSLNz T and CARPD-L1z T or CAR19z T cells post co-cultured with H460-MSLNGL cells [file 40364_2020_198_MOESM1_ESM.pdf]
